# Beyond Vision Restoration: Broadband Retinal Nanoprosthetics with Tellurium Nanowire Networks

**DOI:** 10.34133/research.0857

**Published:** 2025-09-09

**Authors:** Siqi Zhang, Zheng Rong, Liang Chu

**Affiliations:** School of Electronics and Information, Hangzhou Dianzi University, Hangzhou 310018, China.

## Abstract

Retinal degenerative disorders pose critical challenges for vision restoration, with traditional prosthetics limited by spectral sensitivity and invasive surgeries. In recent work published in *Science*, revolutionary tellurium nanowire networks converted broadband visible to near-infrared-II (up to 1,550 nm) light into electrical signals without external bias, restoring functional vision in blind animal models.

The human visual system is one of the most intricate sensory marvels in nature. However, retinal degenerative disorders, including age-related macular degeneration and retinitis pigmentosa (RP), remain great global challenges [1]. Traditional retinal prosthetics, such as photodiode-based implants and optogenetic approaches, have limitations in spectral sensitivity, bulky external devices, and invasive surgeries [2,3]. The human photoreceptor has an inherent inability to detect infrared (IR) light and attempts to restore low-light vision, because of its spectral response range. Human photoreceptors cannot detect IR light due to their limited spectral response range, posing challenges for restoring low-light vision in degenerative diseases. In a recent work published in *Science*, Wang et al. [4] developed a revolutionary retinal nanoprosthesis based on tellurium (Te) nanowire networks (TeNWNs). This device can convert broadband light from the visible (VIS) to near-infrared-II (NIR-II, up to 1,550 nm) spectrum into electrical signals without external bias (Figure 1). The IR sensitivity offers marked benefits, including low-light vision restoration (e.g., navigating dim environments) and potential biomedical applications (e.g., imaging through tissue).

**Fig. 1.**
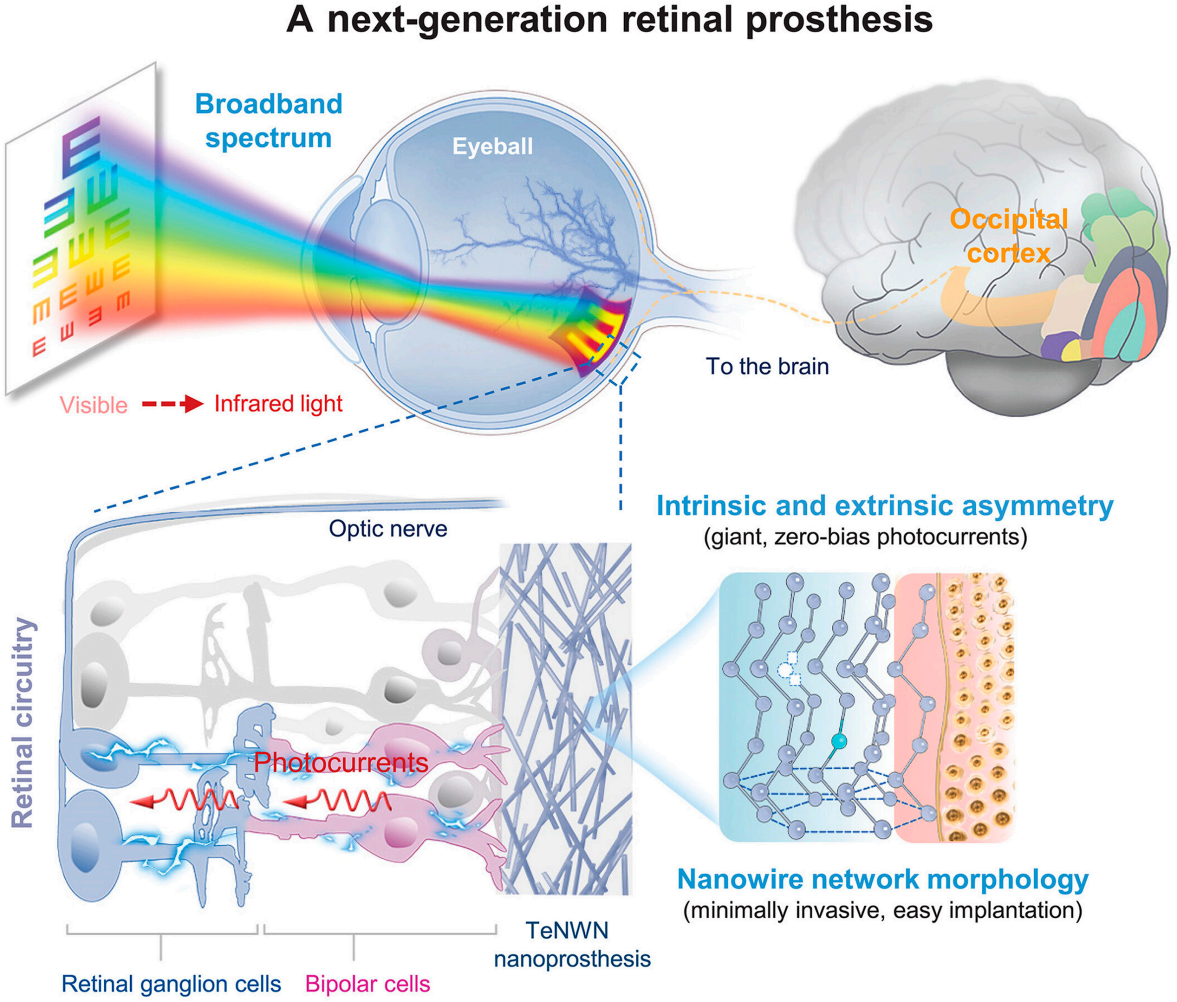
A next-generation nanoprosthesis that restores and enhances vision. Reprinted with permission from Wang et al. [4]. Copyright 2025, *Science*.

The TeNWNs can generate photocurrents across a wide spectral range through material chemistry and structural design. Te with a narrow bandgap (0.3 eV) and high optical absorption coefficient (9% per layer) can detect low-energy photons in the IR range [5]. However, its centrosymmetric crystal structure limits the photovoltaic effect. During chemical vapor deposition, the SnTe_2_ precursor created substitutional Sn defects and Te vacancies within the helical tellurium lattice. These defects break structural symmetry and generate localized electronic states near the Fermi level, which facilitates charge separation and transport. The heterointerfaces formed between TeNWNs and retinal cells, simulated using Au electrodes, created additional asymmetry that drives carrier transport via built-in electric fields.

Te randomly oriented nanowires form porous and interconnected networks (thickness ~150 nm) that simulate the degenerated photoreceptor layers. This design promotes close contact with retinal ganglion cells (RGCs) while minimizing mechanical stress on the retina. The high surface area and vertical alignment relative to the retinal layer of networks ensure efficient light absorption and charge transfer, promoting perpendicular contact with bipolar cells and RGCs to optimize charge transfer. The absence of external bias eliminates the need for intraocular power supplies or extraocular goggles. While polymer nanoparticles also demonstrate photovoltaic properties [6], TeNWNs offer distinct advantages: (a) broader spectral sensitivity (VIS to NIR-II vs. primarily VIS), (b) intrinsic zero-bias operation (eliminating external power), and (c) a porous network structure that minimizes retinal stress compared to bulk nanoparticle injections. Conversely, polymer systems may exhibit better scalability in some fabrication processes. The intrinsic flexibility and biocompatibility of Te nanowires enable minimally invasive subretinal implantation for long-term safety and functionality. This design principle aligns with the “analog compute-in-memory” paradigm [7], thereby reducing energy consumption and complexity. In Pde6brd1/rd1/cDTA mice, a genetic model of RP characterized by complete photoreceptor loss, TeNWNs restored robust neural responses to both VIS (470 to 635 nm) and NIR-II (1,550 nm) light. Action potentials in RGCs showed activation thresholds as low as 18.98 mW mm^−2^ for NIR-II light. The latency of neural responses (200 to 600 ms) and frequency tuning (up to 5 Hz) mirrored those of natural photoreceptors, indicating accurate signal transduction. Visual evoked potential recordings in the occipital cortex further confirmed that TeNWNs generated signals that propagate to higher visual centers [8].

Beyond electrophysiological responses, TeNWNs enabled functional vision recovery in behavioral assays. Implanted blind mice exhibited robust pupil constriction in response to VIS (635 nm) and NIR-II (1,550 nm) light. In contrast, untreated blind mice showed no pupillary light reflex to IR stimulation. To evaluate clinical translatability, the TeNWNs were further implanted into *Macaca fascicularis*. Fundus imaging and optical coherence tomography revealed stable integration with the subretinal space for 112 days, with no signs of retinal detachment or inflammation. Flash electroretinograms displayed robust a- and b-wave responses to NIR-I (940 nm) light (where the a-wave reflects photoreceptor activity and the b-wave originates from bipolar cells), with amplitudes significantly higher in implanted eyes compared to the unimplanted controls. These results confirm the biocompatibility and functional efficacy of the TeNWNs in a primate model, marking a critical step toward human trials.

While TeNWNs indicate a major breakthrough, several technical challenges need to be addressed, including spatial resolution, long-term stability, and spectral tunability [9]. The current receptive field size in implanted mice (135.57 deg^2^) is smaller than that of normal RGCs (163.02 deg^2^), indicating limited spatial discrimination. Increasing nanowire density and the scalability of the arrays could enhance visual acuity; however, trade-offs exist between density and biocompatibility. Although short-term stability (up to 112 days) has been demonstrated in monkeys, the long-term effects of TeNWN degradation or glial scarring remain unclear. Organic–inorganic hybrid coatings or self-healing materials may help mitigate these risks. While broadband sensitivity is advantageous, isolating specific spectral bands (e.g., for color vision) has yet to be addressed. Integrating wavelength-selective filters or layered heterostructures could facilitate spectral multiplexing.

Translating TeNWNs for human use necessitates overcoming substantial clinical and regulatory barriers. Subretinal surgery in humans poses risks of retinal damage and infection. Developing minimally invasive delivery systems, such as injectable nanowire suspensions, may help minimize surgical trauma. Chronic toxicity studies in large animals are crucial for validating long-term biocompatibility. The degradation products of TeNWNs (e.g., tellurium ions) must be demonstrated to be non-toxic or efficiently cleared by the body. Cortical re-education protocols will be vital in assisting patients to interpret prosthetic signals. Neurofeedback training and virtual reality systems may expedite this adaptation process.

In conclusion, TeNWN-based retinal nanoprosthesis signifies a paradigm shift in vision restoration, seamlessly integrating materials science, neuroscience, and biomedical engineering. By leveraging the unique optoelectronic properties of Te and eliminating the external power, this technology addresses long-standing limitations in spectral coverage and invasiveness. The restoration of functional vision in blind mice and primates, accompanied by an extended sensitivity to infrared light, demonstrates a promising therapeutic approach. Like any revolutionary technology, challenges in scalability, long-term safety, and neural adaptation must be addressed. However, the preclinical success of TeNWNs establishes a foundation for human trials, providing hope to millions of patients with untreatable retinal diseases. Beyond therapeutic applications, this work paves the way for augmented vision systems that have the potential to redefine human interaction with the electromagnetic spectrum.
